# Considerations and Indications for Gastric Emptying Scintigraphy in Lung Transplant Patients

**DOI:** 10.22038/aojnmb.2024.80821.1572

**Published:** 2025

**Authors:** Joseph D Sisti, Sean Ide Bolet, Amir Amanullah, Zubair Malik, Henry Parkman, Alan Maurer, Ke Cheng, Simin Dadparvar

**Affiliations:** 1 Temple University Hospital Department of Radiology, Division of Nuclear Medicine and Molecular Imaging, Philadelphia, PA USA; 2 Temple University Hospital Department of Gastroenterology, Philadelphia, PA USA; 3 Department of Biostatistics, Temple University Hospital, Philadelphia, PA USA

**Keywords:** Gastric Emptying Scintigraphy, Lung Transplant Gastroparesis, GES

## Abstract

**Objective(s):**

Gastroparesis is a complication following lung transplantation. This study aimed to assess the prevalence of gastroparesis in patients with lung transplants undergoing solid phase gastric emptying scintigraphy (GES). Specifically, we investigated which type of lung transplant is more susceptible to gastroparesis and whether timing of GES post-transplantation impacts diagnosis of severe gastroparesis.

**Methods:**

This retrospective analysis included lung-transplant recipients between January 2008 and February 2024, who underwent GES. Patients received a standardized egg sandwich labeled with 500 uCi Technetium-99m sulfur colloid. GES results were compared to normal values for percentages retained at 2- and 4-hours post-meal.

**Results:**

Among 485 lung-transplant recipients, 111 (50% male; mean age 63 years) underwent posttransplant GES. Gastroparesis was diagnosed in 23% of lung transplant recipients during the study period. Of those who underwent GES, 67% exhibited delayed gastric emptying, with 38 patients (34%) demonstrating severe retention (>30% at 4 hours). Delayed gastric emptying rates were highest in bilateral lung transplant recipients (73%), followed by left (66%) and right (56%) lung transplant recipients. Timing of GES beyond 6 months or one-year post-transplant did not significantly increase the incidence of delayed gastric emptying (p>0.05). There was no significant difference in proportion of patients with delayed gastric emptying when patients were stratified by gender and age.

**Conclusions:**

Our findings suggest that laterality of lung transplant does not influence risk of delayed gastric emptying. Moreover, early evaluation of gastrointestinal symptoms with GES did not impact the severity or rate of gastroparesis. We recommend routine screening with GES for symptomatic lung transplant recipients, irrespective of transplant timing, to facilitate timely management and reduce post-operative complications associated with gastroparesis.

## Introduction

 Lung transplantation is a critical surgical intervention for end-stage lung diseases with substantial success rates despite associated complications. Per the latest consensus statement by the International Society for Heart and Lung Transplantation, patients meeting specific criteria—namely, facing a mortality risk exceeding 50% without transplantation and demonstrating an estimated 5-year post-transplant survival rate exceeding 80% should be evaluated for lung transplantation (1). 

 Gastroesophageal reflux disease (GERD) and gastroparesis are among the common complications in patients undergoing lung transplantation with some studies reporting post-transplant complication rates for GERD and gastroparesis to be as high as 60% (2). 

 Additionally, an increase in prevalence of esophageal motility disorders ranging from 33.3% to 49.1% after lung transplantation has been reported (3). The etiologies contributing to these complications may involve intraoperative vagal nerve injury and intrathoracic adhesions (2-4). Chronic micro-aspiration has been hypothesized to lead to acute rejection and chronic lung allograft dysfunction (CLAD), subsequently increasing patient morbidity and mortality (5). Despite these findings, other factors potentially exacerbating the prevalence and severity of post-operative complications, such as the specific lung transplanted and the timing of gastroparesis diagnosis via GES, warrant further investigation. 

 GES is currently recognized as the definitive method for diagnosing gastroparesis, evaluating the residual amount of solid food in the stomach at 2- and 4-hour intervals post-ingestion. GES is warranted in patients exhibiting symptomatic gastroparesis, characterized by nausea, vomiting, early satiety, postprandial fullness, bloating, and upper abdominal pain (6).

 In lung transplant recipients, untreated gastroparesis poses significant risks, including heightened susceptibility to gastroesophageal reflux and microaspiration, which can escalate the incidence of transplant rejection and infections (7). Notably, severe gastroparesis may contribute to more severe postoperative complications, such as transplant rejection, due to patient challenges in tolerating immuno-suppressive medications. This intolerance could stem from gastroparesis-related symptoms such as nausea and vomiting, or the delayed gastric emptying hindering adequate medication absorption (8).

 Various treatments have demonstrated potential in alleviating gastroparesis symptoms; however, long-term effectiveness remains elusive for most. In addition to dietary modifications like low-fat, low-calorie regimens, pharmacological interventions encompass prokinetics, antiemetics, and neuro-modulators; however, these medications frequently fall short in addressing the underlying disease mechanisms due to disease progression at the time of diagnosis (9). Recent studies have highlighted per-oral endoscopic pyloromyotomy (POP) as a promising therapeutic approach showcasing significant improvement in delayed gastric emptying with the majority of patients experiencing resolution of symptoms, particularly evident at the 4-hour interval following intervention. This emerging procedure offers potential benefits in managing gastroparesis with implications for long-term symptom relief (10).

 This study aims to assess the frequency of gastroparesis post-lung-transplantation at our high-volume lung transplant center, examine the timing of GES acquisition, and compare gastroparesis rates between recipients of single and bilateral lung transplants. Elucidating these variables in the care of lung transplant recipients can potentially be used to mitigate strain on both patients and healthcare systems grappling with post-operative complications. These findings offer valuable insights for optimizing the management of lung transplant recipients and improving outcomes in this vulnerable patient cohort (11, 12). 

## Methods

 During the study period spanning January 2012 to June 2023, a total of 485 lung transplants were performed at Temple University Hospital in Philadelphia. 111 of these patients (56 male, 55 female) underwent GES post-lung transplantation. Of the 111 post-lung transplant patients undergoing solid phase GES, 33 received a left lung transplant, 27 received a right lung transplant, and 51 received bilateral lung transplants. The predominant underlying pulmonary diseases pre-transplant were pulmonary fibrosis or sarcoidosis in 73 patients (66%) and chronic obstructive pulmonary disease (COPD) in 38 patients (34%). The mean age of patients at the time of transplantation was 63 years, ranging from 28 to 83 years.

 Patients included in this study were those referred for GES post lung transplant at Temple Hospital due to symptoms suggestive of gastroparesis, including nausea, vomiting, dysphagia, belching, abdominal pain, early satiety, and poor appetite (13, 14). Any post-transplant patient who received follow-up care at other hospitals outside of our institution was excluded from the study.

 For GES, patients ingested a standardized egg sandwich meal labeled with 500 microcuries (µCi) of Technetium-99m sulfur colloid, following current Society of Nuclear Medicine recommendations. Stomach images were captured in anterior and posterior projections at 0, 30, 60-, 120-, 180-, and 240-minutes post-meal ingestion using a GE Millennium MG Single Head camera. GES solid phase results were compared against established normal values, with gastric emptying considered abnormal if the residual percentage was greater than 60% at 2 hours and/or greater than 10% at 4 hours post-meal (14).

 Statistical analyses were performed using Chi-Square tests to compare rates of gastroparesis among different lung transplant groups (15). A p-value less than 0.05 was considered statistically significant. Logistic regression models were used to analyze differences in delayed gastric emptying between patients tested within six months versus after one-year post-transplant. Cox regression models were employed to compare mortality rates among patients based on the type of lung transplant received. A p-value less than 0.05 was considered statistically significant.

 The PAGI-SYM score was calculated for 54 patients in this study based on their reported symptoms. Symptomatology corresponding to the PAGI-SYM score, including heartburn/ regurgitation, fullness/early satiety, nausea/ vomiting, bloating, upper abdominal pain, and lower abdominal pain, was recorded for patients undergoing GES (16, 17).

## Results

Among the 111 patients who underwent GES post-lung transplantation, 27 had received a right lung transplant, 33 had a left lung transplant, and 52 underwent bilateral lung transplantation. 

**Figure 1:**
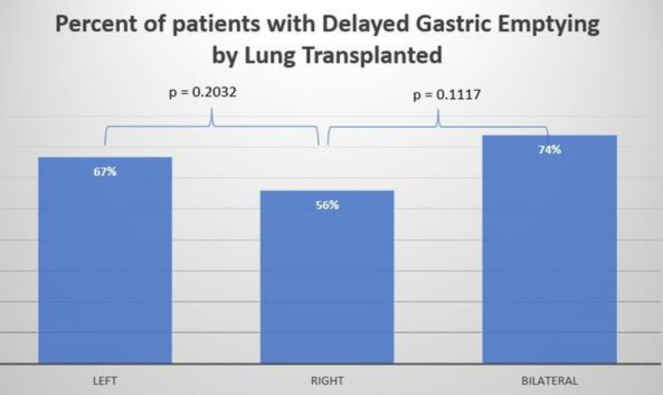
Bar graph comparing incidence of delayed gastric emptying in patients who underwent left lung transplant, right lung transplant, and bilateral lung transplant

 Within this GES cohort, 73 patients (66%) were diagnosed with gastroparesis based on evidence of delayed gastric emptying. The median interval between GES and lung transplant was 131 days. GES procedures were conducted within specific timeframes post-transplantation: 1) within the first 3 months in 28 patients (31%), 2) between 3 to 6 months in 26 patients (29%), 3) between 6 to 12 months in 15 patients (17%), and 4) after more than 12 months in 20 patients (22%). Statistical analysis revealed no significant impact of the timing of GES at 6 or 12 months on the incidence of delayed gastric emptying (p>0.05). Among patients with specific lung transplant types: delayed gastric emptying was observed in 22 out of 33 patients (67%) with left lung transplants, 15 out of 27 patients (56%) with right lung transplants, and 37 out of 51 patients (73%) with bilateral lung transplants, as illustrated in Figure 1.

 56.4% of females (31 out of 55) experienced delayed gastric emptying, while 60.7% of males (34 out of 56) experienced delayed gastric emptying. The chi-square test results in a p-value of 0.785, which indicated no statistically significant difference between males and females regarding the proportion of delayed gastric emptying. Additionally, no significant difference was seen regarding age and proportion of patients with delayed gastric emptying as 62.9% of patients aged 59 and under (22 out of 35) experienced delayed gastric emptying, while 56.6% of patients aged 60 and over (43 out of 76) experienced delayed gastric emptying. The chi-square test for this data yielded a p-value of 0.677, indicating no statistically significant difference between the two age groups in terms of the proportion of delayed gastric emptying.

 The comparison of lung transplants did not reveal a significant difference in the incidence of delayed gastric emptying (see Figure 1). 

**Figure 2:**
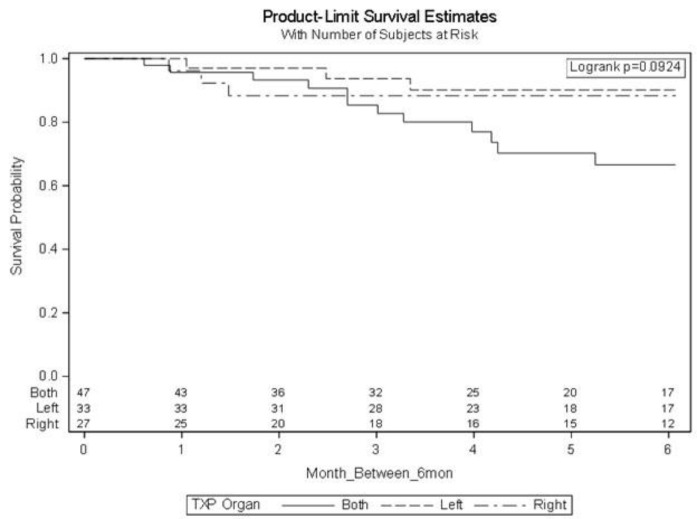
Cox Regression Model demonstrating survival of patients over 6 months by left, right, or bilateral transplant. Values placed above each month on the x axis are remaining patients in each category that have yet to receive a gastric emptying study at the point of time

 Analysis of mortality rates indicated a trend towards increased mortality among patients with bilateral lung transplants at 6 months; however, the sample size was insufficient to establish statistical significance (see Figure. 2). 

**Figure 3:**
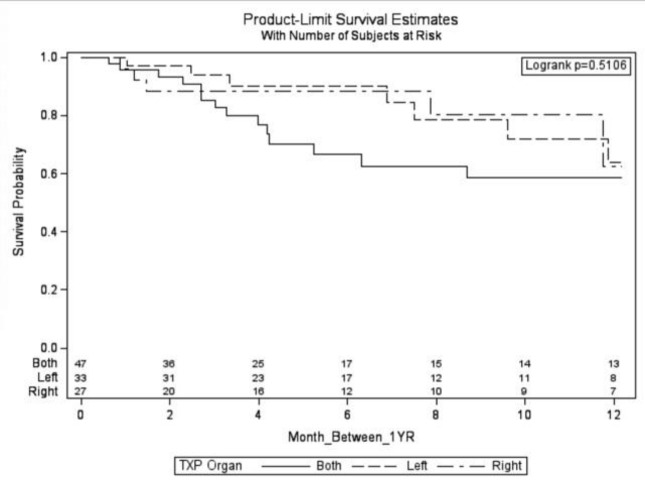
Cox Regression Model demonstrating survival of patients over 12 months by left, right, or bilateral transplant. Values placed above each month on the x axis are remaining patients in each category that have yet to receive a gastric emptying study at the point of time

**Figure 4:**
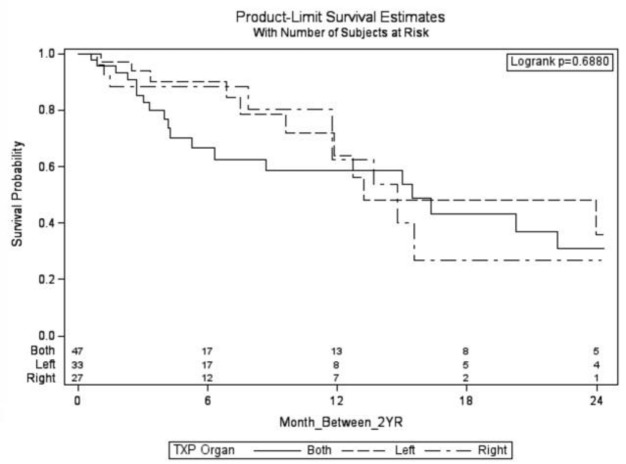
Cox Regression Model demonstrating survival of patients over 24 months by left, right, or bilateral transplant. Values placed above each month on the x axis are remaining patients in each category that have yet to receive a gastric emptying study at the point of time

**Figure 5:**
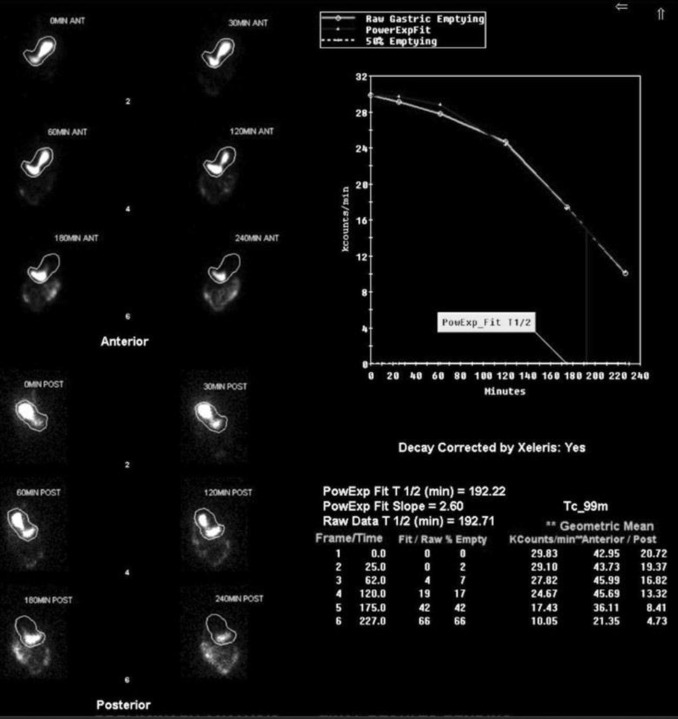
Anterior and posterior GES images for a 63-year-old female who underwent bilateral lung transplant in March 2019 and was diagnosed with severe gastroparesis and delayed emptying at 2 and 4 hours in three months later in June 2019

**Figure 6:**
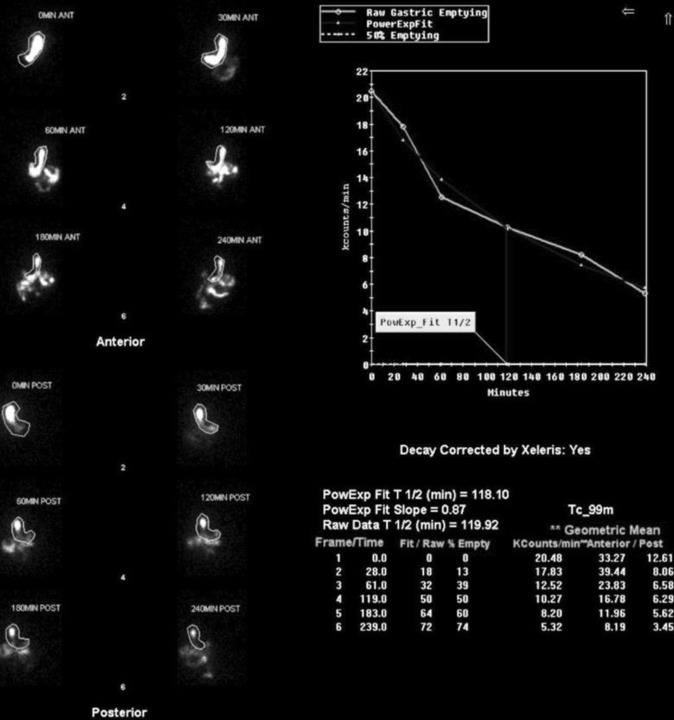
Anterior and posterior GES images for a 73-year-old female who underwent left lung transplant in January 2020 and was diagnosed with severe gastroparesis and delayed emptying at 2 and 4 hours in six months later in June 2020 period

 Notably, outcomes at 12- and 24- month intervals showed similar mortality rates across different lung transplant groups, suggesting that the type of lung transplanted (right, left, or bilateral) did not impact long-term survival nor development of gastroparesis during these timeframes (Figure 3 & 4). GES images are demonstrated by a bilateral lung transplant patient with severe gastroparesis (Figure 5), and a left lung transplant patient with severe gastroparesis (Figure 6).

## Discussion

This study attempts to identify risk factors for gastroparesis in lung transplantation depending on the lung transplanted and whether the severity of gastroparesis coincides with delaying GES in symptomatic patients (18). 

 Among the 485 lung transplant patients considered, 111 underwent GES based on suspicious gastroparesis symptoms, leading to a gastroparesis rate of 23% within the overall cohort. Notably, among the GES-tested patients, 66% were confirmed to have gastroparesis. It is important to acknowledge that the true incidence of delayed gastric emptying among lung transplant recipients may be underestimated due to potential asymptomatic or mildly symptomatic patients who did not undergo GES. Our analysis did not reveal significant differences in delayed gastric emptying rates between patients receiving unilateral versus bilateral lung transplants or between left versus right lung transplants (p=0.11). However, prior studies suggest a higher incidence of gastroparesis following left lung transplantation, likely attributed to the anterior position of the left vagus nerve in the thorax, predisposing it to increased injury risk (19, 20).

 When evaluating the impact of the interval between lung transplantation and the date of GES for diagnosing gastroparesis, our study did not find a significant difference in diagnosis rates between patients tested beyond six months versus one-year post lung transplantation. This suggests that the timing of GES may not substantially affect the detection of gastroparesis in lung transplant recipients. 

 Given that most gastric emptying studies are conducted in response to symptomatic complaints such as early satiety, regurgitation, and nausea, our findings support the notion that gastroparesis manifests primarily due to symptom presentation rather than the duration elapsed since lung transplantation. These results are consistent with current literature emphasizing the importance of close post-transplant follow-up to promptly identify and manage symptoms of delayed gastric emptying, thus mitigating potential complications in lung transplant recipients.

 A recent study has identified an association between gastroparesis and several post-trans

-plantation complications, including Bronchitis Obliterans Syndrome (BOS), Chronic Lung Allograft Dysfunction (CLAD), reflux, and micro aspiration (21). The link between gastroparesis and these conditions may be attributed to bile aspiration causing downregulation of innate immunity, along with impaired cough reflexes and mucociliary clearance leading to aspiration (22). Early diagnosis of gastroparesis using GES when patients present with concerning symptoms could potentially reduce the incidence of these post-transplantation diseases. Timely diagnosis and vigilant surveillance for gastroparesis in symptomatic and asymptomatic post-transplant patients may contribute to decreased morbidity and mortality rates among lung transplant recipients. This underscores the importance of proactive management strategies aimed at addressing gastrointestinal complications following lung transplantation. 

 We hypothesize that the gastric emptying test may progressively worsen over time in patients with vagal nerve injury from transplantation surgery. If validated, earlier detection of gastroparesis could facilitate prompt intervention and potentially improve patient outcomes.

 Furthermore, there is limited research investigating the timing of esophageal dysfunction development post-transplantation. Some studies have suggested that between 3- and 6- months post-transplantation is a common period for the onset of gastroparesis (23). It is important to note that even in the absence of typical gastroparesis symptoms, the discussed complications remain possible. This phenomenon could lead to an underestimation of the incidence of both gastroparesis and delayed gastric emptying in our patient population. Our findings underscore the need for thorough assessment of lung transplant patients for symptoms suggestive of gastroparesis. Appropriate diagnostic testing, including gastric emptying studies, should be considered in symptomatic individuals. 

 However, our study did not identify a definitive timeframe during which patients are at increased risk for symptomatic gastroparesis. Continued research is warranted to better understand the optimal timing and strategies for diagnosing and managing gastrointestinal complications post-lung transplantation.

## Conclusion

 In summary, our study highlights that gastroparesis can manifest at any point following lung transplantation. Although the initial vagus nerve injury during transplantation triggers delayed gastric emptying, patients may exhibit symptoms within weeks or experience delayed complications over years. We advocate for regular follow-up to promptly identify early symptoms and maintain a low threshold for ordering GES, as delayed diagnosis correlates with more severe gastric emptying impairment. Future research should focus on delineating a specific timeline for gastroparesis development post-transplantation. This knowledge could inform more standardized approaches using Nuclear Medicine to manage lung transplant patients, potentially reducing gastrointestinal and GERD symptoms while lowering the incidence of CLAD and BOS (24). 

 Conducting multicenter studies with larger, more diverse patient cohorts would enable better detection of delayed gastric emptying in bilateral or left lung transplant recipients. 

 Additionally, further investigation into symptomatic patients is warranted to assess complications associated with undiagnosed gastroparesis and mitigate potential lung damage post-transplantation.
